# Antidiabetic and Antilipidemic Effect of *Musa balbisiana* Root Extract: A Potent Agent for Glucose Homeostasis in Streptozotocin-Induced Diabetic Rat

**DOI:** 10.3389/fphar.2016.00102

**Published:** 2016-05-02

**Authors:** Himadri Kalita, Dulal C. Boruah, Meetali Deori, Ankita Hazarika, Rahul Sarma, Sima Kumari, Raghuram Kandimalla, Jibon Kotoky, Rajlakshmi Devi

**Affiliations:** ^1^Biochemistry Laboratory, Life Sciences Division, Institute of Advanced Study in Science and TechnologyGuwahati, India; ^2^Department of Botany, Goalpara CollegeGoalpara, India; ^3^Drug Discovery Laboratory, Institute of Advanced Study in Science and TechnologyGuwahati, India

**Keywords:** antioxidant, free radical, crude fiber, hyperglycemia, hyperlipidemia

## Abstract

Folklore studies have revealed that *Musa balbisiana* Colla (MB; Family: Musaceae) has high medicinal properties. The purpose of the present study is to evaluate antihyperglycemic, and antioxidant activity of MB extracts in streptozotocin (STZ) induced diabetic rats. *In vitro* antioxidant and antidiabetic activity of MB extracts, i.e., root extract (RE), shoot extract and inflorescence extract were determined by using various methods viz 1,-1-diphenyl-2-picrylhydrazyl (DPPH) and a method to assess their possible effect on glucose diffusion across gastrointestinal tract and identify bioactive compound of potent extract. *In vivo* antilipidemic and antidiabetic activity was evaluated by administrating oral dose of RE for 15 days on STZ- induced diabetic rat. RE showed highest antioxidant activity by scavenging DPPH radical (IC50 32.96 μg/ml) and inhibit 30% glucose movement *in vitro*. The methanol extract of root showed the presence of calyx [4] arene category of the compound. Furthermore, RE treated rat revealed a reduction in fasting blood glucose (62.5%), serum total cholesterol (36.2%), triglyceride (54.5%), and low-density lipoprotein (50.94%) after 15 days as compared to STZ treated animal. There was an initiation of regenerative structures of the affected organs after 15 days of RE treatment. Histopathological observations clearly differentiate the structural changes in pancreas, liver, and kidney of STZ and RE treated group. The presence of calyx [4] arene class of compound may be responsible for its antioxidant and antidiabetic properties by absorbing glucose *in vivo*.

## Introduction

Diabetes mellitus (DM) is one of the severe endocrine disorder and an important health problem that has been increasing in most of the countries (Mbanya et al., 2010). According to the report of World Health Organization [WHO], (2013) about 347 million people suffer from diabetes worldwide. After cancer and cardiovascular diseases, diabetes has become pandemic to mankind due to its high prevalence, morbidity, and mortality (Egede and Ellis, 2010). According to a new report by the Pharmaceutical Research and Manufacturers of America (PhRMA), American biopharmaceutical companies developed 180 new antidiabetic medicines. These drugs are under development, either in clinical trials or under review by the Food and Drug Administration (FDA). It includes 30 drugs for type 1 diabetes, 100 for type 2 and 52 for diabetes-related conditions. DM is a metabolic disorder characterized by hyperglycemia and insufficiency in secretion and action of endogenous insulin and disturbances of carbohydrate, lipid and protein metabolism (Ansarullah et al., 2011).

A wide range of oral medicines are currently being used considering the major impact of the increasing global prevalence of diabetes due to the absence of efficient and affordable interventions (Inzucchi, 2002). In most of the cases, the antidiabetic medications prescribed by physicians are responsible for various side effects such as liver problems, lactic acidosis, diarrhea and high rates of secondary failures (Inzucchi, 2002). So the traditional system of medicinal plants and practice has been a necessary resource in many countries to control various complications of DM as they are considered to be less toxic and free from side effects than synthetic molecules (Balaraman et al., 2010).

Among the numerous fruit yielding plants, that provide medicinal properties banana is a major fruit, contributing 16% of the world total fruit production (Reported by Food and Agricultural Research, Geneva, 2009). It has been reported that there are 30 different varieties of banana (Family: Musaceae) available in the world (Lehmann et al., 2002). Various parts of the plant can be used for the treatment of various diseases including diabetic, diarrhea, scabies and inflammations as all parts of the plant exhibit different pharmaceutical properties (Rai et al., 2009). To get low-fat, low-sodium, and cholesterol-free diet, the banana is suitable for consumption making it particularly recommendable for people with cardiovascular and kidney problems, arthritis, gout, or gastrointestinal ulcers (Sumathy et al., 2011). Among the different varieties of *Musa* sp., *Musa balbisiana* Colla (MB; Family: Musaceae) is available in South Asia, Southeast Asia, and Southern China. The inflorescence and shoot part of the plant have nutritional properties. The ripe pulp of the MB is widely used as baby food instead of commercially available food for children. From shoot and root part of MB, a food additive is known as “Kolakhar” is prepared which can also be used as soap and detergents for washing cloth, shampooing hair, and antibacterial agent.

Much study has been done regarding the various medicinal properties of different *Musa* sp. It was reported that *M. sapientum* L. (Family: Musaceae) fruits increases insulin secretion by pancreatic β-cells and enhances peripheral glucose utilization, in STZ induced rat (Ojewole and Adewunmi, 2003). Folklore studies revealed that root extract (RE), shoot extract (SE), and inflorescence extract (IE) of MB have high medicinal properties, and the RE is used for the treatment of diabetes (Tribal Healthcare Research Programme, April 2008–March 2012). However, no scientific study has been designed till date regarding the scientific validation of this traditional claim. Therefore an attempt has been made to investigate some pharmacological property of these three extracts of MB with special reference to antidiabetic, antilipidemic, and antioxidant property.

## Materials and Methods

### Chemicals

1,-1-diphenyl-2-picrylhydrazyl (DPPH), trolox, ascorbic acid (AA), thiobarbituric acid (TBA), superoxide dismutase (SOD), catalase (CAT), glutathione (GSH), 5,-5-dithiobis-2-nitrobenzoic acid (DTNB), catechin, quercetin, streptozotocin (STZ) were obtained from Sigma Chemicals (St. Louis, MO, USA). All the other chemicals used in the study were obtained from Merck, India.

### Plant Materials

Plant materials (root, shoot, and inflorescence) were collected from the medicinal plant garden of Institute of Advance Study in Science and Technology (IASST), Guwahati, Kamrup district of Assam, India (situated in between 25°43′–26°53′ North latitude and 90°39′–92°11′ East longitude), in the month from November to April. The plant was authenticated and reconfirmed in the Department of Botany, Gauhati University, Assam. A voucher specimen (IASST/LSD/PM-18) was deposited at the medicinal and aromatic plant section, Life Sciences Department of IASST, Assam.

#### Preparation of Plant Extracts

Root, shoot, and inflorescence were shed dried and grinded into fine powder. Each 1 gm of sample was dissolved in 10 ml of distilled water (1/10, w/v) and kept on a magnetic stirrer for 60 min at 30°C. The solutions obtained were filtered with the muslin cloth and stored at -20°C until use. No preservatives were added.

### Antioxidant Assay

#### DPPH Radical Scavenging Activity

The DPPH (1,-1-diphenyl-2-picrylhydrazyl) scavenging effects of RE, SE, and IE of MB were determined by a standard method (Brand-Williams et al., 1995). In 2.0 ml of each sample, 2.0 ml of 0.16 mM DPPH solution were added. The mixture was vortex for 1 min and kept in the dark at room temperature for 30 min. The absorbance of each sample was measured at 517 nm. The scavenging effect expressed (%) was calculated using the following given Duan’s formula. Commercially available AA was used as the standard.

$$Scavenging\,\;effect(\%)=[1-(A_{sample}-A_{sample\,\;blank}\,)/A_{control}1]\times 100$$

Where the *A*_control_ is the absorbance of the control (DPPH solution without sample), the *A*_sample_ is the absorbance of the test sample (DPPH solution plus test sample), and the *A*_sample blank_ is the absorbance of the sample only (sample without DPPH solution).

#### Hydrogen Peroxide (H_2_O_2_) Radical Scavenging Activity

The radical scavenging activity of the samples against H_2_O_2_ was determined using the method of Ruch et al. (1989). H_2_O_2_ (43 mM) was prepared in 0.1 M phosphate buffer solution (pH 7.4). Samples (1 ml) were mixed with 43 mM hydrogen peroxide solution. After 10 min, the reaction mixture absorbance was measured at 230 nm. The phosphate buffer without hydrogen peroxide was used as blank. Trolox was used as a reference compound.

The inhibition activity was calculated as:

$$Percentage\,\;scavenged\,\;\left(H_{2}O_{2}\,\right)=\left[\left(A0-A1\right)/A0\right]\times 100$$

Where *A*0 is absorbance of the control and *A*1 is absorbance of the samples.

#### Nitric oxide (NO) Radical Scavenging Activity

Nitric oxide radical scavenging activity was carried out as per the method of Sreejayan and Rao (1997). NO radicals were generated from sodium nitroprusside solution. Sodium nitroprusside 1 ml (10 mM) was mixed with 1 ml of RE, SE and IE of MB in 200–1000 μg/ml in phosphate buffer (0.2 M pH 7.4). The mixture was incubated at 25°C for 150 min. After incubation, the reaction mixture was mixed with 1.0 ml of Griess reagent (1% naphthalenediamine dichloride and 2% phosphoric acid). The absorbance was measured at 546 nm and percentage of inhibition was calculated using the above formula. AA used as a standard.

#### Lipid peroxidation (LPO)

Lipid peroxidation induced by Fe^2+^ ascorbate system in rat liver homogenate was estimated as thiobarbituric acid reactive substances (TBARS) by the method of Ohkawa et al. (1979). The reaction mixture contained rat liver homogenate 0.25 ml (10% w/v in 0.05 M phosphate buffer, pH 7.4), 0.1 ml Tris-HCl buffer (150 mM, pH 7.2) and 0.05 ml AA (0.1 mM), 0.05 ml 4.0 mM FeSO4.7H2O and 0.05 ml of RE, SE, and IE of MB. The mixture was incubated at 37°C for 1 h and to it was added 1.5 ml of 0.8% (w/v) 2-thiobarbituric acid, 1.5 ml of 20% acetic acid and 0.2 ml of 8.1% (w/ v) sodium dodecyl sulfate (SDS). Then the volume of the mixture was increased up to 4.0 ml with distilled water and heated at 95°C for 60 min. After cooling with tap water, 1.0 ml of distilled water and 5.0 ml of a mixture of n-butanol and pyridine (15:1, v/v) were added. The mixture was shaken vigorously and centrifuged at 5000 rpm for 10 min. The absorbance of the developed color in the organic layer was measured at 532 nm. Data were expressed as nanomoles per mg tissue mass. Catechin was used as a standard.

### Phytochemical Analysis

#### Total Polyphenol Content

Total phenolic contents of all the three extracts were determined by Folin and Ciocalteu reagent using the method of Hagerman (2002) with slight modifications. All the three extracts (0.5 ml) were mixed with Folin and Ciocalteu reagent (2.5 ml, diluted 10 times) and incubated for 2 min at room temperature followed by addition of sodium carbonate solution (2 ml, 7.5% w/v). The mixture was then allowed to stand for 30 min at room temperature and absorbance was measured at 765 nm. The amount of total polyphenol was calculated as a catechin equivalent from the calibration curve of standard catechin solution and expressed as mg catechin/gm of extract.

#### Total Flavonoid Content

Total flavonoid was estimated according to the method of Jay et al. (1975). RE, SE, and IE, 2 ml of each was mixed with 2 ml of AlCl_3_ in methanol (2%). The absorbance was read at 415 nm after 10 min. Quercetin was used as reference compound, and the result was expressed as mg of quercetin equivalents (QE)/gm of extract.

### Crude Fiber

Crude fiber was estimated following the method of Lanna et al. (2004). Extract (2 gm) was boiled with ether up-to 52°C to remove fat. It was then mixed with 200 ml of sulphuric acid (H_2_SO_4_) for 30 min and remaining was filtered through muslin cloth and washed with boiling water until the complete removal of acid. After washing, it was boiled with sodium hydroxide solution for 30 min. The above solution was again filtered and washes with 25 ml of 1.25% H_2_SO_4_, three 50 ml portions of water and 25 ml alcohol. After removing the residue, it was transferred to ashing dish (pre-weighed dish W1). The residue obtained was then dried for 2 h at 130 ± 2°C and cooled in the desiccators and weigh (W2). It again ignited for 30 min at 600 ± 15°C and cooled in a desiccator and weigh (W3).

% of crude fiber was calculated using the following formula = Loss in weight on ignition (W2–W1) – (W3–W1)/weight of the sample × 100.

### Glucose Movement Studies

A simple model system was used to evaluate effects of plant extracts on glucose movement *in vitro*. This model was adapted from a method described by Edwards et al. (1988), which involved the use of a sealed dialysis tube into which 15 ml of a solution of glucose and NaCl (0.15 M) was introduced, and the appearance of glucose in the external solution was measured. The model used in the present experiment consisted of a dialysis tube 6 cm × 15 mm; (Spectra/P or MWCO: 2000) into which 2 ml of 0.15 M NaCl containing 0.22 mM D-glucose was added. The dialysis tube was sealed at each end and placed in a 50 ml centrifuge tube containing 45 ml of 0.15 M NaCl. The tubes were placed on an orbital shaker (Tarson product) and kept at room temperature (20 ± 2°C). The movement of glucose into the external solution was monitored at set time intervals, as illustrated in the figure. In the experiment, the effects of 60 gm/l plant extracts on glucose diffusion were compared to control test conducted in the absence of plant extract. At the end of the experimental period, the concentrations of glucose within the dialysis tubing were measured. All tests were carried out in triplicate. Glucose concentrations were measured using the glucose oxidase method of analysis.

### GCMS Analysis

The GC – MS analysis was carried out using a GCMS-TQ8030 (Shimadzu Corporation, Kyoto, Japan). An EB-5MS capillary column (30m × 0.25 mm i.d.; 0.25 μm) was used for GC. The instrument was set to a pressure of 57.4 kPa and initial temperature of 50°C and maintained at this temperature for 2.5 min. At the end of this period, the oven temperature was rise to 300°C, at the rate of 15°C/min, and maintained for 8 min. Injection port temperature was ensured at 300°C and Helium flow rate as 1 ml/min. The ionization voltage was 70 eV. The samples were injected in split mode as 20:1. Mass spectral scan range was set at 45–450 (m/z). Compound identification was involved comparison of the spectra with the databases (NIST-11) using a probability based algorithm.

### Preparation of STZ Induced Diabetic Rats

The experiment was conducted using healthy male Wister albino rat (110–150 gm) in accordance with the internationally accepted guideline for experimental animal use and care, and the study was approved by the Institutional Animal Ethics Committee (IAEC; 1706/GO/C/13/CPCSEA). Animals were housed in individual cages in an ambient temperature of 27 ± 3°C and relative humidity 50 ± 5% with a 12 h light-12 h dark cycle. They were fed pellet diet consisting of nitrogen free extract 51.65%, crude protein 21.36%, crude fat 10.63%, total ash 7.41%, moisture 6.32%, crude fiber 2.63%, calcium 1.75%, phosphorous 1.1%, water activity 0.23% per 100 gm of the diet; Nutrilab, Kolkata, India and distilled water.

### Acute Toxicity Evaluation in Mice

Acute oral toxicity study was performed as per OECD 423 guidelines (acute toxic class method), albino mice (*n* = 6) of either sex selected by random sampling were used for acute toxicity study. The animals were kept fasting overnight and provided only with water, after which the RE was administered orally at 5 mg/kg and observed for 15 days. If mortality is observed as two out of three animals, then the dose administered is assigned as a toxic dose. If mortality observed in one animal, then the same dose is repeated to confirm the toxicity study. If mortality is not observed, the procedure is repeated for higher doses such as 1000, 2000, and 3000 mg/kg.

### Animal Treatment

Diabetes was induced by a single intraperitoneal (i/p) injection of STZ (55 mg/kg in 0.1 M citrate buffer, pH 4.5; Srinivasan et al., 2005). Blood glucose concentration and changes in body weight were regularly monitored. After 3 days, rats with diabetes having hyperglycemia [i.e., with fasting blood glucose (FBG) of 250–400 mg/dl] were taken for the experiment. Three different doses were selected based on the traditional claim, i.e., 50, 100, and 200 mg/kg of RE for the animal experiment. Among them, 100 mg/kg was found to be most effective. Hence, this dose has been selected for the present study. The rats were divided into four groups of six rats each as follows:

Group A: Received only normal pellet diet and water *ad libitum*.
Group B: STZ (55 mg/kg) treated hyperglycemic group. (Srinivasan et al., 2005).
Group C: Extract treated (100 mg/kg) group.
Group D: Glibenclamide (10 mg/kg) treated group (Gupta et al., 2012).

At the end of the stipulated period (15 days), the animals were sacrificed after overnight fasting and blood were collected from jugular vein (Tiwari et al., 2014) and serum was separated by centrifugation. Pancreas, liver and kidneys were isolated, washed with ice-cold saline and preserved for biochemical and histopathological analysis.

#### Long-Term Effect of RE on FBG, Body Weight and Water Intake

Fasting blood glucose, body weight and water intake were measured on day 1st, 5th, 10th, and 15th day of the experiment. FBG was measured by using glucometer (Accu-Check Active Roche, Germany).

#### Metabolic Parameters

Total cholesterol (TC), serum triglyceride (TG), and high-density-lipoprotein-cholesterol (HDL-c) were determined using enzymatic kits (Crest Biosystems, Goa). Low-density-lipoprotein-cholesterol (LDL-c) and very low-density lipoprotein- cholesterol (VLDL-c) were calculated using Friedwald’s formula (Friedewald et al., 1972).

#### Endogenous Oxidants and Oxidative Stress Markers

Endogenous antioxidants, such as GSH by Ellman (1959); SOD by Marklund and Marklund (1974) and CAT by Góth (1991) were measured. TBARS was measured as a marker of LPO by using the procedure described by Ohkawa et al. (1979). Nitrate/nitrite levels were measured by using the standard method described by Green et al. (1982).

#### Total Antioxidant Activity (TAA)

The antioxidant activities were measured with a Photochem system (Analytik Jena AG, The Woodlands, TX, USA). The system enables the quantification of antioxidant capacity of blood serum and liver homogenate based on photo chemiluminescence (PCL). This includes photochemical excitation to generate free radicals (superoxide anion radicals) followed by luminescence detection method Popov and Lewin (1999). The free radicals generated by the optical excitation of the photosensitizer substance were partly eliminated by the reaction of antioxidants in the sample to be analyzed. In a measurement cell, the luminescence of the detection material (luminol) generated by the remaining radicals is measured and thus the quantity of antioxidants present in the sample was determined in equivalents to AA. PCL can measure antioxidant activity in the nano molar range whereas other antioxidant assays determine activity in the micro molar range. The reagent kits (ACW) used for the analysis were obtained from Analytik Jena AG. 10 μl for serum and 2 μl of liver homogenate were used for each measurement.

#### Pathological Histology

For histopathological analysis of pancreas, liver and kidney, samples were collected from all groups and fixed in 10% buffered formalin. After routine processing, the tissues were embedded in paraffin by using Leica Histopathology Assembly (Leica TP 1020 and Leica EG 1150 H), sectioned at 5 μm and stained with routine haematoxylin-eosin (H-E) in Leica Autostainer XL. After staining images were obtained under Phase contrast microscope (Leica).

### Statistical Analysis

All data are presented as mean ± SEM. *In vitro* antioxidant values of RE, SE, and IE was tested using one-way analysis of variance. Data from group A, B, C, and D were tested by two-way analysis of variance followed by Bonferroni’s post hock test. All statistical analysis were performed using Graph Pad Prism version 5 for Windows. The *P*-value of <0.001 was considered as statistically significant.

## Results

### DPPH and H_2_O_2_ Free Radical Scavenging Activity

The three extracts showed a concentration dependent scavenging activity against DPPH and H_2_O_2_ radicals. IC_50_ values of the RE, SE, and IE against DPPH free radical was found to be 32.96, 42.4, and 38 μg/ml, respectively, which could be comparable with the AA with an IC_50_ value of 36.6 μg/ml. In the case of H_2_O_2,_ the IC_50_ values were 57.6, 36.8, and 40.8 μg/ml, respectively, while that of trolox showed an IC_50_ value of 42.8 μg/ml.

### LPO and NO Inhibition Assay

In the case of LPO, IC_50_ values of RE, SE, and IE were found to be 23.96, 39.09, and 44.88 μg/ml, respectively, and that of catechin is 25.99 μg/ml. The NO radical also scavenged by three extracts and their 50% inhibitions were 33.99, 45.09, and 39.77 μg/ml, respectively, and the AA was 30.56 μg/ml.

### Phytochemical Analysis

#### Total Polyphenol and Flavonoid Content of RE, SE, and IE of MB

Total polyphenol content quantified in RE, SE, and IE were 3.88 ± 0.0057, 0.69 ± 0.00057, and 2.51 ± 0.032 mg catechin/gm of extract, respectively. Flavonoid content of RE, SE, and IE were 0.24 ± 0.015, 0.80 ± 0.006, and 0.065 ± 0.0085 mg quercetin/gm of extract, respectively. Among the three extracts, RE showed highest amount of polyphenol followed by IE and SE.

### Crude Fiber in RE, SE, and IE

The amount of crude fiber in RE, SE, and IE were 4.01 ± 0.00, 4.01 ± 0.001, and 2.50 ± 0.001 gm/100 gm of the whole extract, respectively. RE have the highest amount of crude fiber as compared to SE and IE.

### Effect of RE, SE, IE on Glucose Diffusion

After 30 h without plant extract (control), glucose movement out of dialysis tube had reached maximum level with a mean glucose concentration in the external solution was 0.16 ± 0.009 mg/ml. RE of MB was the most potent inhibitor of glucose movement in the model system with external glucose concentration 0.099 ± 0.004 mg/ml after 30 h. In the case of SE and IE, the concentration of glucose in the external medium after 30 h was found to be 0.1 ± 0.01 and 0.14 ± 0.01 mg/ml, respectively.

### GC-MS Analysis

**FIGURE 1:**
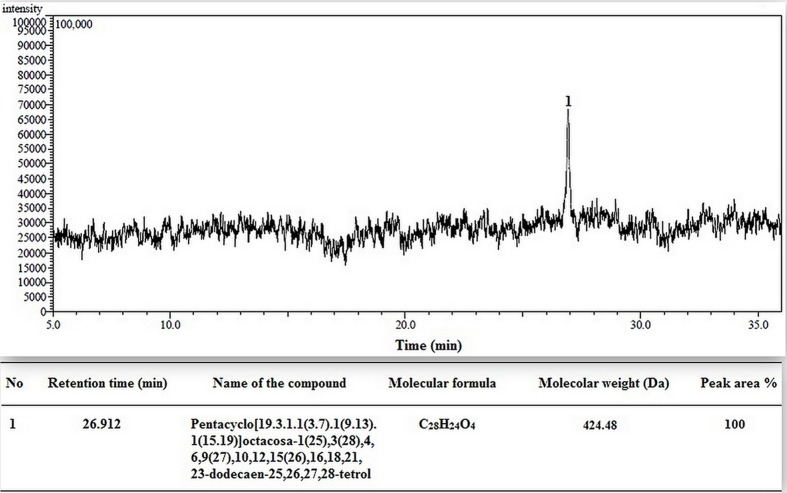
**Typical GC-MS chromatogram of *Musa balbisiana* methanolic root extract.** Major compound identified as (1) Pentacyclo[19.3.1.1(3.7).1(9.13).1(15.19)] octacosa1(25),3(28),4,6,9(27),10,12,15(26),16,18,21,23-dodecaen-25, 26, 27, 28-tetrol.

After GC-MS analysis of methanol extract of RE, different compound were identified, but among them the compound which showed highest concentration was Pentacyclo[19.3.1.1(3.7).1(9.13).1(15.19)]octacosa-1(25),3(28),4,6,9(27),10,12,15(26),16,18,21,23-dodecaen-25, 26, 27, 28-tetrol (**Figure 1**).

### Acute Toxicity Effect

The results of the acute oral administration of RE of MB in various doses of 1000, 2000, and 3000 mg/kg indicated no mortality up to 15 days after treatment.

During the experimental period of 15 days, four animals died in Group B, Group C, and Group D after STZ treatment.

### Long-Term Effect of RE on FBG

**FIGURE 2:**
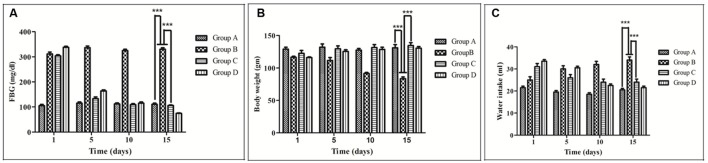
**Effect of *Musa balbisiana* root extract on different parameters.** **(A)** Fasting blood glucose (FBG) **(B)** Body weight changes **(C)** Water intake. All the results were expressed in mean ± SEM (n = 6); ^∗∗∗^*p* < 0.001 in comparison between different groups.

Fasting blood glucose level significantly increased about 62.5% in case of group B compared to group A. Treatment of group D with glibenclamide restored blood glucose to almost normal level. Interestingly, a similar effect was observed in rats administrating RE of MB (group C) at a dose of 100 mg/kg (**Figure 2A**).

### Effect of RE on Body Weight and Water Intake

Body weight of group B was decreasing day by day from the 1st to 15th day (131–111.67 gm) of the experiment. On the contrary, it has been found that the group C animals were gaining their weight from 111.67 g to 133.33 gm (**Figure 2B**). Water intake of group B was increasing day by day, and the amount was much higher (55 ± 5 ml) than the group A. In the case the of group C it has been found that the water intake volume (24 ± 5.77 ml) became less as compared to group B (**Figure 2C**)

### Effect of RE on Serum Lipid Profile

Serum lipids (TC, TG, VLDL-c, and LDL-c) were increased significantly (*p* < 0.05) in diabetic animals in comparison to that of Group A (**Table 1**). However, these parameters were decreased significantly (*p* < 0.01) in the group C (about 54.9% for VLDL-c and 50.94% for LDL-c). HDL-c, a beneficial lipoprotein, was decreased in group B as compared to that of group A, and the result reversed in group C (*p* < 0.01; **Table 1**).

**Table 1:** Changes in levels of serum lipid profile (TC, TG, HDL-c, LDL-c, and VLDL-c) and oxidative stress markers (SOD, GSH, Catalase, TBARS, and NO) in serum and liver in groups A, B, C, and D.

Parameters	Group A	Group B	Group C	Group D
**Serum lipid profile**				
TC (mg/dl)	70.34 ± 1.9	186.23 ± 34.13^+^	119.36 ± 6.01**	111.1 ± 4.1
TG (mg/dl)	120 ± 0.86	149.40 ± 8.28	127.54 ± 7.63*	129.29 ± 7.61
HDL-c (mg/dl)	37.66 ± 0.89	21.90 ± 2.58	30.33 ± 5.55	34.33 ± 10.12
LDL-c (mg/dl)	56.76 ± 1.49	194.21 ± 5.57^++^	114.53 ± 3.40*	102.62 ± 7.5
VLDL-c (mg/dl)	24 ± 0.17	29.88 ± 5.6	25.50 ± 3.45	25.85 ± 3.45
**Oxidative stress markers**				
Serun SOD (% SOD)	33.52 ± 1.34	22.19 ± 3.00^+^	48.79 ± 8.9**	48.66 ± 0.57
Serum GSH (ug/ml)	1300.67 ± 49	1134.66 ± 54.45	2552 ± 579.75	2873.33 ± 148.4
Serum catalase (unit mg protein)	152.71 ± 2.4	44 ± 6.55^++^	112.06 ± 17.52**	115.53 ± 12.78
Serum TBARS (nmole/ml)	28.7 ± 2.23	37.7 ± 4.66	23.24 ± 6.07	20.56 ± 4.56
Serum NO (uM/ml)	23.19 ± 4.11	30.92 ± 5.89	25.03 ± 2.33	24.29 ± 3.56
Liver SOD (% SOD)	68 ± 0.89	32.29 ± 10.39^+^	57.71 ± 3.03	61.65 ± 6.24
Liver GSH (ug/gm)	1361.33 ± 120.3	896 ± 148.1^++^	1364.66 ± 30.74	1416.66 ± 104
Liver catalase (unit mg protein)	26.12 ± 2.42	17.50 ± 2.42^+^	39.02 ± 1	42.03 ± 2.61
Liver TBARS (nmole/mg tissue)	31.23 ± 2.4	50.3 ± 1.48^++^	22.85 ± 0.18	24.91 ± 4.96
Liver NO (uM/mg)	23.92 ± 5.67	40.91 ± 3.44	28.61 ± 6.11	27.60 ± 5.55


*^∗^*p* < 0.05 vs. diabetic control, ^∗∗^*p* < 0.01 vs. diabetic control, ^+^*p* < 0.05 vs. control, ^++^*p* < 0.01 vs. control. *n* = 6, mean ± SEM.*

### Effect of RE on Endogenous Antioxidants and Oxidative Stress Markers

Lipid peroxidation level was elevated significantly in group B compared to group A. Administration of RE (100 mg/kg) reduced the LPO level significantly on the 15th day (**Table 1**). The GSH level was found to be low in the liver and serum of group B (*p* < 0.01) while in the group C the levels increased on the 15th day when compared with the untreated diabetic animals (*p* < 0.01; **Table 1**). The SOD activity was found to be reduced in the liver and serum of animals treated with STZ (*p* < 0.01). SOD values in rats treated with STZ along with RE was significantly higher (*p* < 0.01) on the 15th day (**Table 1**). In liver and serum of diabetic rat, NO level was found to be more as compared to that of control (**Table 1**). RE and glibenclamide treated groups showed a lower level of NO as compared to the diabetic control group.

### TAA of Serum and Liver

The TAA in the serum and liver of group A, group B, group C, and group D were quantified in equivalents to AA. Serum TAA in group A, group B, group C, and group D were 0.95 ± 0.0049, 0.1 ± 0.14, 1.09 ± 0.27, and 1.6 ± 2.0 nmol/ml, respectively. Liver TAA in group A, group B, group C, and group D were 2.3 ± 0.05, 0.71 ± 0.7, 2.2 ± 0.28, 2.5 ± 0.21 nmol/mg of tissue, respectively. A high TAA was observed in the liver and serum of group C as compared to group B.

### Histopathological Changes

#### Pancreas

**FIGURE 3:**
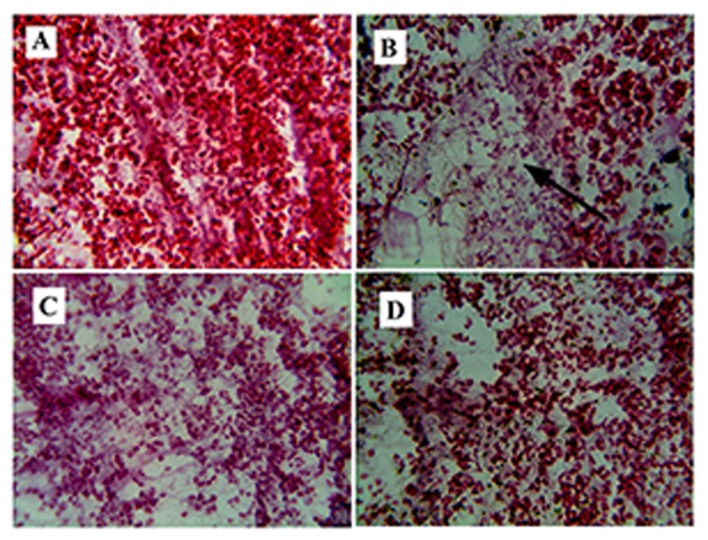
**Fibrosis in pancreas.** **(B)** Represents Hematoxylene Eosine staining of streptozotocin (STZ) induced pancreatic section, showing fibrosis in the β-cells of pancreas (represented by arrow). **(A)** Represents normal pancreas with acinar cells. **(C)** It is the root extract (RE (100 mg/kg) treated pancreatic section, showing improvement in pancreatic structure and **(D)** represents glibenclamide (10 mg/kg) treated pancreatic section showing reversal of the normal pancreatic structure.

HE-stained tissue sections of group B elicited severe injury of pancreatic β-cells, such as the decrease of the islet cells numbers, damage of acinar cells (**Figure 3B**) as compared to group A (**Figure 3A**). Group D (glibenclamide, 10 mg/kg) showed a significant effect on reducing injuries of the pancreas (**Figure 3D**). The damage in the pancreas was partially reversed in group C (**Figure 3C**).

#### Liver

**FIGURE 4:**
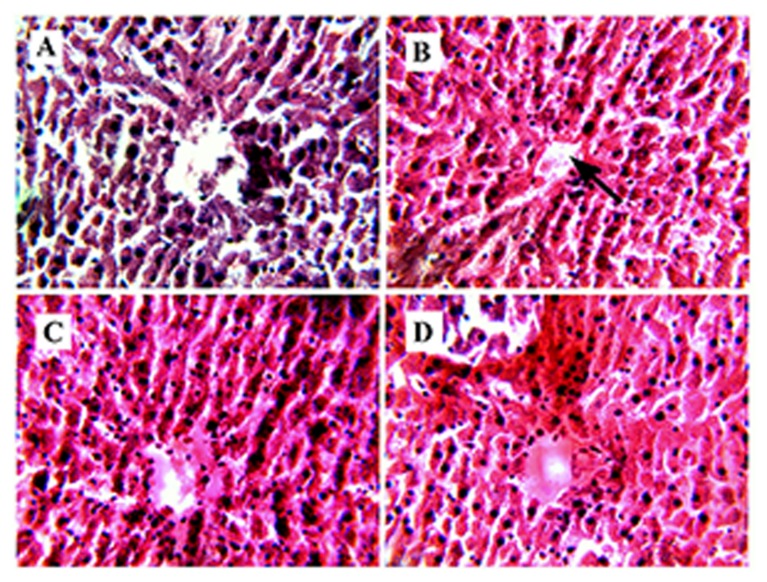
**Congested central vein.** **(B)** Represents Hematoxylene Eosine staining of STZ induced liver section, showing congestion in central vein (represented by arrow). **(A)** represents normal sized central vein. **(C)** It is the RE (100 mg/kg) treated liver section, showing improvement in the hepatic cell structure and **(D)** represents glibenclamide (10 mg/kg) treated liver section, showing development in the hepatic structure.

In liver tissue degeneration of hepatocytes, dissolved cytoplasm, necrosis, congestion in central vein and intrahepatic hemorrhages were observed in group B (**Figure 4B**). After the administration of RE of MB (group C) severe hepatic lesions induced by STZ were markedly reduced (**Figure 4C**). Group B (**Figure 4D**) showed mild to moderate intrahepatic hemorrhages and few erythrocytes in the central vein as compared to group A (**Figure 4A**).

#### Kidney

**FIGURE 5:**
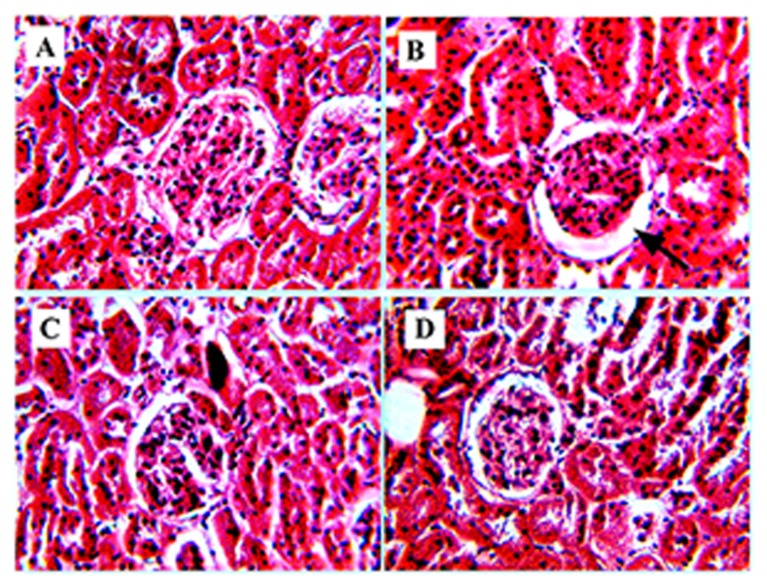
**Increase in bowman’s space of kidney.** **(B)** Represents Hematoxylene Eosine staining of STZ induced kidney section, showing wider bowman’s space (represented by arrow). **(A)** Represents normal kidney with glomerulus. **(C)** It is the RE (100 mg/kg) treated kidney, represents improvement in the glomerular structure and **(D)** represents glibenclamide (10 mg/kg) treated kidney section, showing restoration of the bowman’s space of glomerulus.

Wider bowman’s space, severe hemorrhages in glomeruli, mild intertubular hemorrhages, and mild dilatations were observed in group B (**Figure 5B**). The kidney of group C showed mild intertubular hemorrhages and restoration of glomeruli with few erythrocytes (**Figure 5C**). Group D showed moderate hemorrhages in glomeruli (**Figure 5D**) in comparison to group A (**Figure 5A**).

## Discussion

*Musa balbisiana* Colla has high medicinal properties, which is used for the treatment of diabetes, cardiovascular disease, inflammation, etc., from time immemorial in different parts of the world without any scientific basis. For this reason, we have screened out the different extracts of MB for its antioxidant, antilipidemic and antidiabetic activity. In this study, RE of MB shows a high antioxidant activity than that of SE and IE. RE scavenged DPPH, H_2_O_2_, NO radicals and caused inhibition of LPO in an efficient way. Antioxidant plays a significant role in scavenging highly reactive free radicals. Reactive oxygen species (ROS) such as H_2_O_2_, superoxide (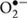) and hydroxyl (.OH) radicals are responsible for OS at the time of diseased condition, as supported by increased cellular accumulation of lipid peroxides and depletion of endogenous antioxidants (Dhalla et al., 2000). OS in diabetes coexists with a reduction in the antioxidant status, which can increase the deleterious effects of free radicals. In diabetes, OS may occur due to auto-oxidation of glucose, shifts in redox balances, decreased tissue concentrations of low molecular weight antioxidants, such as reduced glutathione (GSH) and impaired activities of antioxidant defense enzymes such as SOD, CAT, and increase MDA level in the body (Haskins et al., 2003). ROS generated by high glucose is causally linked to other metabolic abnormalities important to the development of diabetic complications. Besides this, RE was the most potent inhibitor in glucose movement tested in an *in vitro* glucose diffusion model. The *in vitro* glucose diffusion model employed constant agitation to mimic gastrointestinal convection (Edwards et al., 1987). This inhibition of glucose movement of RE may be due to the presence of complex carbohydrate. Dietary fiber which is the part of plant material includes different types of complex carbohydrate can influence gastrointestinal physiology and also the absorption and metabolism of carbohydrate and fat. The fiber present in RE may be responsible for absorption and metabolism of carbohydrate; hence, the concentration of glucose outside the dialysis tube was less as compared to that of SE and IE.

The chromatogram for methanol extract of root showed the presence of Pentacyclo[19.3.1.1(3.7).1(9.13).1(15.19)]octacosa1(25),3(28),4,6,9(27),10,12,15(26),16,18,21, 23-dodecaen-25, 26, 27, 28-tetrol with retention time 26.91 min]. This compound is a derivative of calyx [4] arene class of compound, which act as metal chelating agent and conjugate with Zn^2+^ or Mg^2+^ and perform its activity (Pathak et al., 2011).

Administration (i/p) of STZ reduced the body weight of the experimental animals significantly within 3 days, which was retained after 15 days of RE treatment in group C (*p* < 0.001). Derangement of metabolic pathways leads to decrease in body weight, and it is a common feature of diabetes (Al-Shamaony et al., 1994). The loss of body weight in STZ induced diabetes might be due to the increased muscle waste and loss of tissue proteins (Swanston-Flatt et al., 1990). When blood glucose levels are particularly high, some of the excess glucose pass into the urine, which causes excess urination (reported by Diabetes Learning Centre, 2006). Excess urination leads to increase in water intake in case of STZ treated animal and on RE treatment, the amount of water intake reduce to normal level in group C (*p* < 0.001; **Figure 2C**). Moreover, the RE of MB at a dose of 100 mg/kg showed significant improvement in FBG in STZ treated hyperglycemic rat. Enhancement of glucokinase (GK) activity and inhibition of glucose-6 phosphatase (G6pase) and phosphoenolpyruvate carboxykinase (PEPCK) in the liver may be responsible for the anti-hyperglycemic action of RE. Reduction in the blood glucose may be the result of hepatic GK expression, which can stimulate the residual pancreatic mechanism, probably by accelerating the utilization of blood glucose for energy production or glycogen storage in the liver. A decrease in G6pase and PEPCK activities, on the other hand, indicates a decrease in hepatic glucose production, since they are the key enzymes involved in the regulation of gluconeogenesis and glucose output from the liver (Son et al., 2011).

In diabetic condition, coronary artery disease is the most common cause of death. Lipid abnormalities are commonly associated with diabetes, particularly in those with type 2 diabetes (American Family Physician, 1999). Hyperglycemia leads to hypertriglyceridemia due to overproduction of TG-rich lipoproteins in the liver, associated with decreased HDL-c and increase cholesterol levels (Abbate and Brunzell, 1990). In STZ induced rats after RE treatment the TG, TC, LDL-c were significantly decreased, and the level of HDL-c increased than that of diabetic rats. Based on the results, RE seems to exert cholesterol lowering effects. The results derived from the present study due to RE treatment suggested that it possesses not only hypoglycemic property but also have hypolipidemic effect in STZ induced hyperlipidemic rats.

Induction of OS due to depletion of antioxidant scavenger system is the diabetogenic properties of STZ, mediated by pancreatic β-cell destruction thereby resulting in an elevated level of LPO that is a clear manifestation of the promotion of *de novo* free radicals generation leading to tissue damage (Kaleem et al., 2006). The significant decrease in the LPO level after RE treatment in diabetic animals indicates that RE effectively enhanced the antioxidant potential *in vivo*, by acting as a strong 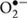 and singlet oxygen (^1^O_2_)quenchers. Tissue GSH plays a central role in antioxidant defense by detoxifying ROS directly or in a glutathione peroxidase-catalyzed mechanism (Ramesh and Pugalendi, 2006). The significant decrease in GSH content in diabetic animals was might be due to increasing utilization of GSH against toxic effects of LPO, free radicals. RE, by directly scavenging the free radicals in diabetic rats reduced the utilization of GSH, thereby resulting in an increased GSH content. Endogenous antioxidant enzymes (SOD and CAT) are responsible for the detoxification of deleterious oxygen radicals (Ramesh and Pugalendi, 2006). In this experiment, the SOD and CAT activities were decreased in diabetic rats which could be due to increase utilization for scavenging free radicals. Treatment with RE and glibenclamide has reversed the activities of these enzymatic antioxidants which could be a result of decreased LPO and decreased utilization. NO synthase synthesized NO has been implicated in a variety of pathologies including diabetes. Aldose reductase is regulated by NO that is known to cause hyperglycemia (Srivastava et al., 2005). After RE treatment, NO level is reduced to the normal level which is comparable to that of the diabetic-induced group.

Photo chemiluminescence assay is an analytical method that is rapid, relatively simple, and reproducible, making it an attractive bio monitoring tool especially for food supplement, nutrition and food technologies. This study is based on the TAA, expressed as the sum of the hydrophilic antioxidant capacity referred to a common reference compounds, AA. This value resulted to be a useful index to describe the capacity of complex samples of natural origin, to counteract ROS and, in particular, the 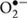 radical, very harmful to human health (Schlesier et al., 2002). TAA both in the case of liver and serum, the diabetic-induced group shows the lowest amount as compared to that of control. In the case of RE and glibenclamide treated groups the values of TAA were much higher than that of diabetic animal, this might be because of the protection of different antioxidant enzymes like SOD, GSH, CAT as reported in the results.

Increase in the FBG and lipid levels, which was seen in the experiment, may be responsible for the structural changes of the pancreas in STZ treated rat (**Figure 3A**). Pancreatic stellate cells activate the synthesis of free fatty acids (FFAs) and lipid peroxide. These stellate cells help in the progression of pancreatic fibro genesis (Fernandes-Santos et al., 2009). Fibrosis occurs in the extracellular matrix, in which area acinar cells disappear. The treatment of RE, reduced the progression of fibrosis, thereby protecting the pancreas from further damage (**Figure 3C**).

Histopathological studies of diabetic liver section showed a degenerative structure with central vein congestion and cellular swelling (**Figure 4B**). ROS, which was generated from STZ, may be responsible for all these hepatic damages (Ananthan et al., 2004; Hazarika et al., 2016). However, diabetic rats treated with RE can restore the normal uncongested central vein (**Figure 4A**). This proves that RE can protect the hepatic structure from OS generated by STZ.

Moreover, histology of the kidney in diabetic rats showed widening of bowman’s space area, severe hemorrhages in glomeruli, intertubular hemorrhages and mild dilatation (**Figure 5B**). Diuresis is a common feature associated with diabetes, which may lead to the structural changes observed in glomerulus (Shanmugasundaram et al., 1983). Enlargement of bowman’s space is uncertain, but it may be due to glomerular hyperfiltration rate (GFR; Henegar et al., 2001). These observed pathological changes were decreased in RE treated diabetic rats (**Figure 5C**). The glomeruli appear to be normal with mild hemorrhages, which may be due to the protective effect of RE. RE may decrease the GFR, which in turn may responsible for narrowing of the bowman’s space of glomerulus.

From this study, it is inferred for the first time that the RE of MB is a good candidate and complementary medicine in the management of diabetes mellitus. The results of the present study indicate that the RE is capable of exhibiting significant antihyperglycemic activities in STZ induced diabetic rat by improving parameters like water intake, body weight, TAA, antioxidant enzyme and lipid profile as well as regeneration of pancreatic islets of Langerhans, hepatic structures. The presence of Pentacyclo[19.3.1.1(3.7).1(9.13).1(15.19)]octacosa1(25),3(28),4,6,9(27),10,12,15(26),16,18,21,23-dodecaen-25,26,27,28-tetrol a calyx[4]arene class of compound in RE may be responsible for the antioxidant properties, which in turn may be partially responsible for its antidiabetogenic and antilipidemic properties.

## Author Contributions

HK conceived and designed the experiment. HK, MD, AH, SK, RK, and RS performed the experiment. HK and DB analyzed the data. HK wrote the manuscript. DB, RD, and JK have done critical revision of the manuscript for important intellectual content. RD has been the corresponding author throughout the writing process. All authors have contributed to the final version and approved the final manuscript.

## Conflict of Interest Statement

The authors declare that the research was conducted in the absence of any commercial or financial relationships that could be construed as a potential conflict of interest.
